# QTL Mapping and Candidate Gene Analysis for Pod Shattering Tolerance in Soybean (*Glycine max*)

**DOI:** 10.3390/plants9091163

**Published:** 2020-09-08

**Authors:** Jeong-Hyun Seo, Beom-Kyu Kang, Sanjeev K. Dhungana, Jae-Hyeon Oh, Man-Soo Choi, Ji-Hee Park, Sang-Ouk Shin, Hong-Sik Kim, In-Youl Baek, Jung-Sook Sung, Chan-Sik Jung, Ki-Seung Kim, Tae-Hwan Jun

**Affiliations:** 1 National Institute of Crop Science, Rural Development Administration, Jeonju 55365, Korea; next0501@korea.kr (J.-H.S.); hellobk01@korea.kr (B.-K.K.); sanjeev@korea.kr (S.K.D.); mschoi73@korea.kr (M.-S.C.); heeya91@korea.kr (J.-H.P.); shinso32@korea.kr (S.-O.S.); kimhongs@korea.kr (H.-S.K.); baekiy@korea.kr (I.-Y.B.); sjs31@korea.kr (J.-S.S.); jung100@korea.kr (C.-S.J.); 2 National Institute of Agricultural Sciences, Rural Development Administration, Jeonju 55365, Korea; jhoh8288@korea.kr; 3 FarmHannong, Ltd., Daejeon 34115, Korea; leehan26@snu.ac.kr; 4 Department of Plant Bioscience, Pusan National University, Miryang 50463, Korea; 5 Life and Industry Convergence Research Institute, Pusan National University, Miryang 50463, Korea

**Keywords:** soybean, pod shattering, quantitative trait loci, candidate gene, abscisic acid

## Abstract

Pod shattering is an important reproductive process in many wild species. However, pod shattering at the maturing stage can result in severe yield loss. The objectives of this study were to discover quantitative trait loci (QTLs) for pod shattering using two recombinant inbred line (RIL) populations derived from an elite cultivar having pod shattering tolerance, namely “Daewonkong”, and to predict novel candidate QTL/genes involved in pod shattering based on their allele patterns. We found several QTLs with more than 10% phenotypic variance explained (PVE) on seven different chromosomes and found a novel candidate QTL on chromosome 16 (*qPS-DS16-1*) from the allele patterns in the QTL region. Out of the 41 annotated genes in the QTL region, six were found to contain SNP (single-nucleotide polymorphism)/indel variations in the coding sequence of the parents compared to the soybean reference genome. Among the six potential candidate genes, *Glyma.16g076600*, one of the genes with known function, showed a highly differential expression levels between the tolerant and susceptible parents in the growth stages R3 to R6. Further, *Glyma.16g076600* is a homolog of *AT4G19230* in *Arabidopsis*, whose function is related to abscisic acid catabolism. The results provide useful information to understand the genetic mechanism of pod shattering and could be used for improving the efficiency of marker-assisted selection for developing varieties of soybeans tolerant to pod shattering.

## 1. Introduction

Pod shattering is an essential mechanism for reproduction under appropriate environments in many wild species [1,2,3]. Several morphological and physiological changes have been found during domestication, and this is termed ‘domestication syndrome’ [4,5]. Among the domestication syndromes, pod shattering is one of the key strategies for increasing the survival rate of plants through reducing competition [4]. Crops including soybean (*Glycine max*) have been evolved to become more tolerant to pod shattering than their wild progenitors through the natural selection from non-pod shattering plants [6].

Pod shattering is common in the species of Legume, Gramineae, and Brassicaceae crop families [2,7]. In addition, many studies on pod shattering have been conducted in domesticated crops, resulting in numerous genes being identified in rice [8,9], soybean [2,10], common bean [11], *Medicago* [12], and cowpea [13,14]. In soybean, pod shattering at maturing stages can result in serious yield loss, especially under dry weather conditions. The cultivation of pod-shattering-susceptible cultivars could lead to yield losses of 50–100% [15]. In many breeding programs, tolerance to pod shattering is considered an essential trait to minimize yield loss under current environmental conditions, including global warming and dry weather conditions.

Pod shattering in soybeans is considered a quantitative trait controlled by several genes [16] and researchers have reported that the trait is regulated by a major quantitative trait locus (QTL) and multiple minor QTLs [17]. In soybeans, a major QTL associated with pod shattering was first identified on chromosome 16 using restriction fragment length polymorphism (RFLP) markers [18]. Funatsuki et al. [19] found this QTL to be localized between the simple sequence repeat (SSR) markers Sat_093 and Sat_366 and named it *qPDH1* [20]. Several studies detected the *qPDH1* locus through linkage mapping using bi-parental populations with different genetic backgrounds [16,21,22,23,24]. Fine mapping [25,26] led to cloning of the *Pdh1* (*Glyma.16g141400*) gene, which increases the torsion of dried pods under low humidity, and was found to have a premature stop codon [2]. Also identified using wild soybean was the *NAC* gene, named *SHAT1-5* (*Glyma.16g019400*), which activates secondary wall biosynthesis and promotes the thickening of fiber cap cells [10]. Several minor QTLs were also detected on chromosomes 2, 5, 10, 14, and 19 [16,18]. More recently, several novel QTLs were found on chromosomes 1, 4, 6, 8, 9, 11, 17, 18, and 20 through genome-wide association analysis (GWAS) [27], on chromosomes 1, 5, 8, and 14 through specific-locus amplified fragment sequencing (SLAF) [28], and on chromosome 19 through RNA sequencing [5]. Despite the high value of phenotypic variance explained (PVE) by the major QTL on chromosome 16 (*qPDH1*), which accounts for more than 50% of the entire phenotypic variance [19,23], the mechanism of pod shattering tolerance is, presently, still not completely understood. Therefore, additional QTL information from diverse genetic backgrounds is pivotal to developing soybean cultivars with shattering tolerance.

Daewonkong (DW) [29], developed in 1997, is an elite cultivar with several desirable traits, such as strong tolerance to pod shattering and seed quality for processing soy food, and accounts for more than 80% of the total soybean cultivation area in Korea [30]. Although it is an important genetic source for pod shattering tolerance in Korea, genetic information on the pod shattering tolerance of DW has not previously been reported. In our previous study [30], we investigated pod shattering tolerance using two recombinant inbred line (RIL) populations derived from DW and found that the lines that were tolerant and susceptible to pod shattering were remarkably distinct. From the results, we predicted that a major QTL would be segregated in the populations.

The objectives of this study were (1) to construct high-density linkage maps and discover the QTLs for pod shattering using two RIL populations derived from DW and (2) to predict novel candidate QTL/genes based on the allele patterns obtained in the populations. To achieve these objectives, we evaluated the pod shattering ratio in the RIL populations over three years and genotyped the populations using a 180 K SNP array.

## 2. Results

### 2.1. Variation in Pod Shattering Ratios

**Figure 1:**
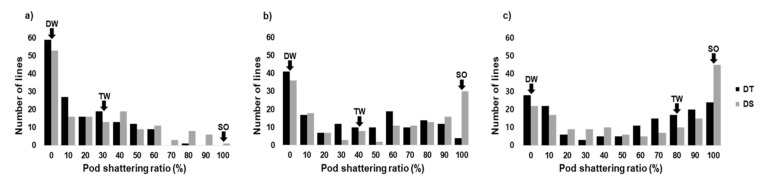
Distribution of pod shattering in the two recombinant inbred line (RIL) populations after pod drying; (**a**) for 24 h, (**b**) for 48 h, (**c**) for 72 h (DW; Daewonkong, TW; Tawonkong, SO; Saeolkong) (Seo et al. 2019).

To evaluate the pod shattering tolerance of the mapped populations, we performed the oven-dry test for soybean grown over a three-year period (2016 to 2018) and calculated the pod shattering ratio after drying matured pods for 24, 48, and 72 h [30]. Across the three experimental years, DW showed a strong tolerance to pod shattering with no shattered pods in the three oven-dry tests. On the other hand, all the pods of Saeolkong (SO) were shattered after 24 h in the oven-dry test. Tawonkong (TW) showed a moderate tolerance up to 24 h with a 25% pod shattering ratio. However, up to 80% of the pods were shattered after 72 h of the drying test (Table 1 and Figure 1).

In the DT population, 16.6%, 37.0%, and 49.7% of pods were shattered after 24, 48, and 72 h, respectively, showing a tendency for more pod shattering with the extension of drying time. A similar tendency was also found in the DS population with 26.7%, 48.7%, and 55.3% pod shattering ratios after 24, 48, and 72 h, respectively (Table 1). The *H^2^* in the DT population ranged from 0.38 to 0.88 and, in the DS population, from 0.62 to 0.84 (Table 1). The distributions of pod shattering in the mapping populations were similar (Figure 1).

### 2.2. Linkage Map Construction

Out of the 169,028 high-quality and genotyped SNP markers, 24,407 (DW and TW) and 21,462 (DW and SO) markers were found to be polymorphic between the parental lines. After deleting the redundant markers with >5% missing values and segregation distortion of *p* < 0.05, a total of 2321 and 1739 SNPs were selected and used to construct linkage maps for the DT and DS populations, respectively. The SNPs were distributed across all 20 chromosomes with 1.1 and 1.8 cM average distances between adjacent SNPs in the DT and DS populations, respectively (Supplementary Tables S1 and S2).

### 2.3. QTL Analysis

Across the years of the experiment, a major QTL was detected on chromosome 16 in both populations with high logarithm of odds (LOD) (up to 67.1 in DT and 60.0 in DS) and PVE values (up to 82.8% in DT and 80.1% in DS). The major QTL identified in the present study was designated as *qPS-DT16-2* and includes the *qPDH1* locus (Table 2 and Table 3). Similarly, *qPS-DT16-1* and *qPS-DS16-3* were also detected within the *qPDH1* locus. These QTLs were stably detected in individuals and across the years. The LOD scores for the QTLs of the DT population ranged 13.4–67.1 with a PVE of 26.1–82.8%, and those of the DS population ranged 14.6–60.0 and 36.0–80.1%, respectively (Table 2 and Table 3). Specifically, *qPS-DT16-2* showed the highest LOD and PVE values in 2017 for 72 h of drying. The identified QTL region contains five candidate genes which are located within the previously reported *qPDH1* locus [21,23,26].

**Figure 2:**
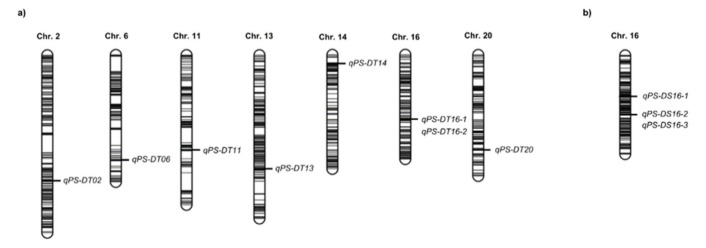
Quantitative trait loci (QTLs) associated with pod shattering tolerance; (**a**) population derived from ‘Daewonkong’ and ‘Tawonkong’, (**b**) population derived from ‘Daewonkong’ and ‘Saeolkong’. The bars inside each chromosome represent the position of markers used to construct the linkage map.

Several population-specific QTLs for pod shattering were also detected on chromosomes 2, 6, 11, 13, 14, and 20 in the DT population and on chromosome 16 in the DS population (Figure 2). Possibly, due to the great effect of the major QTL, these QTLs showed relatively lower LOD (3.6 to 33.4) and PVE (1.3 to 16.4%) values than those of the major QTL. *qPS-DT13* showed the lowest PVE value and *qPS-DT14* showed the highest PVE value. Among these QTLs, *qPS-DT11*, *qPS-DT14,* and *qPS-DS16-1* were also promising QTLs with relatively high values of PVE (>10%). All QTLs showed a negative additive effect (Table 2 and Table 3).

### 2.4. Phenotypic Variation According to the Allele Patterns

**Figure 3:**
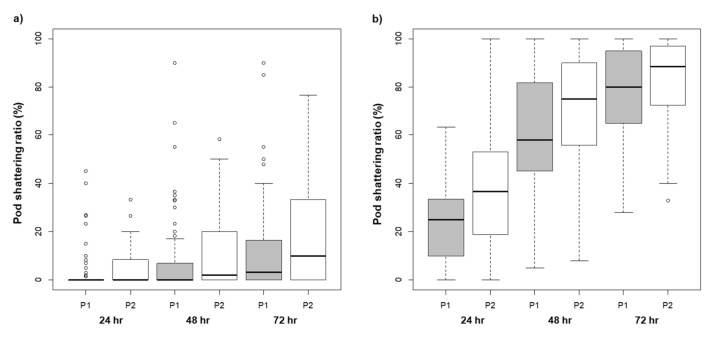
Variation of pod shattering ratio after 24, 48, and 72 h of drying according to the allele patterns in *qPS-DS16-2* (major QTL locus) and *qPS-DS16-1* locus. ‘P1’ indicates that the allele patterns are the same as ‘Daewonkong (DW)’ and ‘P2’ indicates that the allele patterns are the same as ‘Saeolkong (SO)’; (**a**) following ‘P1’ allele in *qPS-DS16-2*, (**b**) following ‘P2’ allele in *qPS-DS16-2*.

The combination of RILs with the P1 (DW) allele in *qPS-DS16-2* (major QTL locus) and *qPS-DS16-1* simultaneously presented stronger tolerance to pod shattering with a 2.5% pod shattering ratio compared to those with the P2 (SO) allele (6.2%, *p* < 0.05) at 24 h of drying. Despite having the P2 (SO) allele in *qPS-DS16-2*, the RILs with the P1 (DW) allele in *qPS-DS16-1* presented better tolerance than those with the P2 (SO) allele at all drying periods (Table 4). There were also differences in the pod shattering ratio according to the allele patterns in the *qPS-DS16-1* region; RILs in which the P1 (DW) allele followed the *qPS-DS16-1* region showed a higher level of tolerance to pod shattering than when the P2 (SO) allele followed (Figure 3). However, *qPS-DT11* and *qPS-DT14* showed no significant differences in pod shattering tolerance regardless of the P1 (DW) or P2 (TW) allele in the *major QTL (qPS-DT16-2)* region (Table 4). Considering all allele patterns of the QTL, the *qPS-DS16-1* region played a key role in the pod shattering tolerance of the RIL populations. Therefore, we inferred the existence of a candidate gene for pod shattering tolerance in the *qPS-DS16-1* region.

### 2.5. Identification of Candidate Genes within and Near QTL

In the present study, several QTLs were detected on chromosome 16. Within and near the QTL regions, there were five candidate genes in the major QTL (*qPS-DT16-2*) which showed the highest value of PVE and 41 candidate genes in the promising QTL *(qPS-DS16-1)* (Table 2 and Table 3). Among the genes residing in the promising QTL regions, six genes had SNPs or insertions/deletions that could cause amino acid sequence variations: *Glyma.16g072700*, *Glyma.16g073700*, *Glyma.16g076100*, *Glyma.16g076300*, *Glyma.16g076500*, and *Glyma.16g076600* (Table 5).

**Figure 4:**
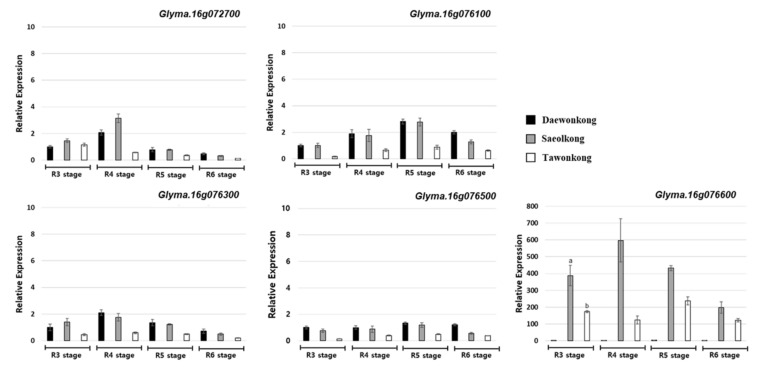
Expression level of five candidate genes in the pods harvested at the growth stages from R3 to R6. Same letters denote no significant differences at 0.05 by DMRT.

Except for *Glyma.16g073700* (unknown function), the expression of the other five genes (Supplementary Table S3) was analyzed by qPCR in the pods harvested from R3 (beginning pod) to R6 (full seed) growing stages [31,32]. Among these five genes, *Glyma.16g076600* showed a significantly different expression level between the tolerant parent (DW) and susceptible parents (TW and SO) (Figure 4). There were six polymorphisms in *Glyma.16g076600*, which includes four missense variant SNP and two insertion/deletion. In particular, an 18 bp insertion in position 7,775,970 is a stop-gain mutation that results in a premature termination codon at the end of the sequence in exon 7.

## 3. Discussion

Pod shattering at the maturing stage (R8, full maturity) is an essential strategy for reproduction in wild species, including in *Glycine soja*. However, it can cause tremendous yield loss in cultivated crop species and be one of the main limiting factors for machinery harvesting. Therefore, developing varieties tolerant to pod shattering is one of the fundamental goals of many crop breeding programs. In Korea, DW is the most widely cultivated soybean cultivar due to its strong tolerance to pod shattering. Therefore, DW could be a valuable genetic source for developing pod shattering tolerant varieties. In the present study, DW showed strong tolerance to pod shattering in the oven-dry test, where no pods were shattered until 72 h of drying (Supplementary Figure S1), whereas for the susceptible parents, TW and SO, numerous pods were shattered after 24 h of drying (Table 1 and Figure 1).

In soybean, pod shattering is considered as a quantitative trait of high heritability that is controlled by a major gene and several minor genes [17]. Since identification of the first major QTL on chromosome 16 [18], two genes on chromosome 16 have been characterized, named *pdh1* (*Glyma.16g141400*) from cultivated soybean and *SHAT1-5* (*Glyma16g019400*) from wild soybean [2,10]. In cultivated soybean, *pdh1* is a major gene for pod shattering and is annotated as a dirigent-like protein in the soybean reference genome (Wm82.a1.v2). Recently, based on the changed gene model Wm82.a2.v1, the *pdh1* gene was identified as *Glyma.16g141400* (unknown function). In this study, we exploited the distinct phenotypic difference between the parents to successfully identify the major QTL on chromosome 16 located within or nearby the *qPDH1* locus in both populations (*qPS-DT16-1*, *qPS-DT16-2*, *qPS-DS16-2*, and *qPS-DS16-3*) (Table 2 and Table 3). However, the QTL region including *SHAT1-5,* which was identified in previous studies, was not detected in the present study. Among the major QTLs, the *qPS-DT16-2* in the DT population showed the highest LOD and PVE values in this study. The QTL region presented higher PVE values (82.8%) and was mapped at a narrower interval (59 kb) than previous studies, and the region was found to have only five candidate genes (Table 2 and Table 3). The negative values of the additive effect indicate that the phenotypic variation explained by the QTLs result from the pod shattering-tolerant parent DW.

More recently, several novel candidate genes were identified on chromosome 9 (*Glyma09g06290*) using genome-wide association mapping [27], chromosome 1 (*Glyma.01g045800* and *Glyma.01g046000*), chromosome 5 (*Glyma.05g005600*, *Glyma.05g225900*, and *Glyma.05g227400*), and chromosome 8 (*Glyma.08g271900* and *Glyma.08g274500*) by specific-locus amplified fragment sequencing [28], and chromosome 19 (*Glyma.19g231900*) by RNA sequencing [5]. In this study, we also found several QTLs that showed relatively low PVE values on chromosomes 2, 6, 11, 13, 14, 16, and 20 (Figure 2). Among these, only *qPS-DT02* on chromosome 2 mapped to a previously reported region, in a study by Kang et al. (2009), whereas the others mapped to different physical positions on the same chromosomes [16,27,28] (Table 2 and Table 3). There was a total of 472 candidate genes in novel QTLs based on the physical position of Williams82 (Wm82.a2.v1). Those genes were related to many metabolic and biological process in plant and contributed to biosynthesis or catabolism of several plant hormones such as auxin, gibberellic acid, and abscisic acid (ABA) (Supplementary Table S4).

Among the detected QTLs, excluding the *qPDH1* locus, we selected only the promising QTLs for further searching of candidate genes with >10% PVE values on chromosomes 11, 14, and 16. The results of the allele patterns of these QTLs showed that the allele patterns in the *qPS-DS16-1* region could lead to a significant difference in the pod shattering tolerance between genotypes. A total of 41 candidate genes were annotated within or near the *qPS-DS16-1* region (72–78 Mbp, based on the physical position of the reference genome Wm82.a2.v1) (Table 3). Out of the 41 genes, six genes have SNP/indel variations in the coding sequence of the parents. *Glyma.16g076600*, one of the six genes, showed highly differential levels of expression between the parents in the growth stages R3 to R6 (Figure 4). This gene was highly expressed in the susceptible parent SO, one of the parents of the RIL population where QTL *qPS-DS16-1* was detected. On the other hand, there was a very low expression of this gene in the resistant parent DW and intermediate expression in TW, indicating the role of *Glyma.16g076600* in pod shattering.

*Glyma.16g076600* is a homolog gene of *AT4G19230* in *Arabidopsis*, which is a member of the CYP707A gene family encoding a protein related to ABA catabolism [33]. ABA is a hormone that responses to environmental stresses and regulates water consumption in plants. Under drought conditions, the endogenous ABA level increases in plants, and the enzyme CYP707A controls endogenous ABA levels; overexpression of CYP707A leads to a decrease in ABA levels [34,35]. An upregulation of ABA induces the expression of many genes that play an important role in the adaptation to abiotic stresses and reduced shattering [36].

In this study, the distinctly high expression of *Glyma.16g076600* in TW and SO could be an indicator of the level of ABA production in the pods that might cause pod shattering. The role of hormones, including ABA, has been reported as regulating silique dehiscence in *Arabidopsis* and *Brassica* [36]. Thus, it could be assumed that the *Glyma.16g076600* regulates the ABA expression in pods, and high expression could be associated with susceptibility to pod shattering. However, further studies are required to confirm the relationship between the pod shattering and ABA levels in the pods as well as other parts in soybeans.

## 4. Materials and Methods

### 4.1. Plant Material

Two RIL populations were derived from the crosses between a pod-shattering-tolerant cultivar Daewonkong (DW) as the female parent and two pod-shattering-susceptible cultivars, Tawonkong (TW) [37] and Saeolkong (SO) [38], as the male parents. Daewonkong, developed in 1997, is the most popular soybean cultivar in South Korea due to its strong tolerance to pod shattering. The RIL populations were developed through the single seed descent method from the F_2_ generation to F_5:6_, F_6:7_, and F_7:8_ generations in each experimental year (2016, 2017, and 2018). The populations consisting of 154 lines (DW × TW (DT)) and 153 lines (DW × SO (DS)) were used to construct high-density linkage maps and to analyze for QTLs associated with pod shattering tolerance [30].

### 4.2. Evaluation of Pod Shattering Tolerance

The evaluation of pod shattering tolerance over three years was performed as described in our previous study [30]. Briefly, mature pods from a total of 307 RILs and three parents grown in field conditions at the Miryang in South Korea were harvested at the maturing stage (R8 stage) and kept at room temperature for a week [23]. After one week, the pod shattering ratio of each RIL was determined as the ratio of the number of shattered pods to the total number of pods counted for 24, 48, and 72 h of drying in a dry oven at 40 °C [39] for three experimental years (2016, 2017, and 2018).

### 4.3. Genomic DNA Extraction and Genotyping

Young trifoliate leaves of the parents and F_7:8_ RILs were bulk harvested at the V2 stage and kept in a deep freezer at −80 °C. The bulked leaves were ground using a bead beater (TissueLyser II; Qiagen, Hilden, Germany). The genomic DNA was extracted using a commercial kit (Exgene Plant SV Miniprep Kit; GeneAll, Seoul, Korea) following the manufacturer’s instruction and 50 µL of AE buffer was used to elute DNA [40]. The 180K Axiom^®^ SoyaSNP array [41,42,43] was used to genotype the parents and 307 RILs of two populations.

### 4.4. Linkage Map Construction and QTL Mapping

Prior to the construction of linkage maps, redundant markers with identical segregation patterns that did not provide an additional contribution to genetic mapping were discarded using the Bin function in QTL IciMapping V4.2 software. The parameters for map construction were set as grouping by a 3.0 logarithm of odds (LOD) threshold, ordering by nnTwoOpt, and rippling by the sum of adjacent recombination fractions. Mapping was conducted using Kosambi’s mapping function, and QTLs were identified with inclusive composite interval mapping (ICIM) using QTL IciMapping V4.2 [44].

The names of QTLs were designated by combining different letters and numbers as follows: *q*, quantitative trait loci, *PS*, pod shattering, *DT*, RIL derived from the cross between Daewonkong and Tawonkong, and *DS*, RIL derived from the cross between Daewonkong and Saeolkong. The numbers followed by the letters indicating the name of the RIL population signify the chromosome containing the QTL. For example, QTLs named *qPS-DT16-2* and *qPS-DT11* indicate the second QTL for pod shattering on chromosome 16 and only one QTL for pod shattering on chromosome 11 in the RIL population (DT) derived from Daewonkong and Tawonkong, respectively.

### 4.5. Prediction of Novel Candidate QTL and Genes

The QTLs with high PVE values (>10%), excluding the *qPDH1* locus, were selected. Then, the statistically significant QTLs for pod shattering were identified according to their allele patterns based on the SNP sequence within the QTL regions. Candidate genes within or adjacent to the candidate QTL were identified based on the SoyBase (www.soybase.org) and Phytozome (www.phytozome.net). The gene description was obtained from the Williams_82 soybean reference genome (Wm82.a2.v1).

### 4.6. Gene Expression Analysis of the Candidate Genes

For total RNA extraction, pods of DW, TW, and SO were harvested from bulked plants at the R3, R4, R5, and R6 growth stages, respectively. The total RNA of the pods was isolated using a RNeasy PowerPlant Kit (Qiagen, Hilden, Germany) and the cDNA was synthesized using a reverse transcription reaction (EcoDry cDNA Synthesis Premix, Takara Bio, Inc., Ohtsu, Japan) following the manufacturer’s instructions. Gene expression was determined by qPCR using an ABI 7300 system (Applied Biosystems, Foster City, CA, USA) with Power SYBR Green PCR Master Mix (Applied Biosystems, Woolston Warrington, UK). All experiments were performed with three replications, and the results were analyzed using ExpressionSuite Software V1.3 (Life Technologies, Foster City, CA, USA). Primers for the target genes (Supplementary Table S3) were designed using Primer3.0 (http://primer3.ut.ee/). *GmActin* gene (*Glyma.18g290800*) was used as a control gene.

### 4.7. Statistical Analysis

Student’s t-test and Duncan’s multiple range test (DMRT) were conducted using R V3.6.3 software (R Core Team, 2020) to compare the phenotypic variations of pod shattering tolerance and the expression levels of candidate genes. The broad-sense heritability (*H*^2^) was calculated using the following equation: $H^{2}=\;\sigma_{g}^{2}\;/\;\sigma_{p}^{2}$ [45].

## 5. Conclusions

In the present study, one major QTL, on chromosome 16, which included the *qPDH1* locus, and several novel QTLs on chromosomes 2, 6, 11, 13, 14, 16, and 20 were identified from high-density linkage maps of two RIL populations. Among these novel QTLs, we identified *qPS-DS16-1* (*Glyma.16g076600*) as potentially playing a role in pod shattering tolerance based on the expression patterns of this gene. The function of *Glyma.16g076600*, a member of the CYP707A family, has been reported as possibly being related to the catabolism of ABA, a hormone known to be associated with a variety of physiological functions, including pod shattering. Therefore, the results of this study provide useful information to understand the genetic mechanism of pod shattering and could be used for improving the efficiency of marker-assisted selection for developing pod-shattering-tolerant varieties in soybean.

## Figures and Tables

**Table 1:** Mean pod shattering ratio of the parents and recombinant inbred lines (RILs) in two mapping populations over three years.

Drying Time (Hour)	Year	Pod Shattering Ratio ± SE (%) ^†^					Broad-Sense Heritability	
		Parents			RILs			
		DW	TW	SO	DT	DS	DT	DS
24	2016	0	25	100	12.1 ± 24.6	24.4 ± 33.5		
	2017	0	0	100	9.9 ± 34.8	23.3 ± 33.8		
	2018	0	50	100	27.8 ± 40.4	32.5 ± 32.0		
average		0	25	100	16.6 ± 19.0c ^‡^	26.7 ± 26.2c	0.38	0.62
48	2016	0	35	100	27.8 ± 34.7	47.7 ± 42.9		
	2017	0	20	100	38.4 ± 39.6	48.0 ± 46.1		
	2018	0	50	100	44.8 ± 36.4	50.5 ± 38.6		
average		0	35	100	37.0 ± 31.9b	48.7 ± 39.1b	0.78	0.84
72	2016	0	80	100	43.6 ± 40.3	54.7 ± 43.6		
	2017	0	100	100	53.5 ± 43.2	51.1 ± 46.8		
	2018	0	60	100	52.0 ± 37.7	60.1 ± 37.3		
average		0	80	100	49.7 ± 37.4a	55.3 ± 39.0a	0.88	0.82

^†^ mean pod shattering ratio at the different drying time over three experimental years (DW; Daewonkong, TW; Tawonkong, SO; Saeolkong, DT; RIL derived from crossing between ‘Daewonkong’ and ‘Tawonkong’, DS; RIL derived from crossing between ‘Daewonkong’ and ‘Saeolkong’. ^‡^ Different letters mean their means are significantly different in the Duncan’s multiple range test (*p* < 0.05).

**Table 2:** Quantitative trait loci (QTLs) significantly associated with pod shattering tolerance in the recombinant inbred line (RIL) population derived from ‘Daewonkong’ and ‘Tawonkong’.

Name of QTL	Chr. (LG) ^1^	Year	Drying Time (Hour)	Position (cM)	Physical Interval ^2^ (bp)	LOD ^3^	PVE ^4^ (%)	Add ^5^	No. Candidate Genes	References
*qPS-DT02*	2 (D1b)	2017	24	122	7,211,739–8,082,789	6.0	1.9	−5.9	87	Kang et al. 2009
*qPS-DT06*	6 (C2)	2016	48	103	5,498,397–6,170,213	3.6	6.1	−9.0	82	Novel
*qPS-DT11*	11 (B1)	2017	48	92	26,717,840–30,547,921	17.4	13.3	−20.0	189	Novel
*qPS-DT13*	13 (F)	2017	24	108	35,019,516–35,459,889	4.3	1.3	−5.0	54	Novel
*qPS-DT14*	14 (B2)	2017	24	11	48,063,838–48,101,634	33.4	16.4	−17.8	2	Novel
*qPS-DT16-1*	16 (J)	2017	24	63	29,532,807–29,809,243	7.4	2.3	−6.7	21	*qPDH1*(nearby)
		2018	72		29,532,807–29,809,243	31.0	67.8	−32.0		
*qPS-DT16-2*	16 (J)	2016	48	64	29,913,393–29,972,096	13.4	26.1	−19.0	5	*qPDH1* (Funatsuki et al. 2008)
		2016	72		29,913,393–29,972,096	23.9	48.0	−29.2		
		2017	48		29,913,393–29,972,096	30.1	29.6	−30.3		
		2017	72		29,913,393–29,972,096	67.1	82.8	−42.0		
		Combined ^6^	24		29,913,393–29,972,096	13.4	33.1	−11.2		
		Combined	48		29,913,393–29,972,096	35.7	60.8	−25.9		
		Combined	72		29,913,393–29,972,096	55.4	78.7	−34.6		
*qPS-DT20*	20	Combined	48	93	2,151,689–2,392,383	3.7	3.7	−6.3	17	Novel

^1^ Chromosome (Linkage group). ^2^ Based on the physical position of ‘Williams82’ ver. 2.1 (www.soybase.org). ^3^ Logarithm of odds. ^4^ Phenotypic variance explained by QTL. ^5^ Additive effect; negative values indicate that ‘Daewonkong’ contributed to the trait. ^6^ Average value of 3 years, 2016, 2017, and 2018.

**Table 3:** Quantitative trait loci (QTLs) significantly associated with pod shattering tolerance in the recombinant inbred line (RIL) population derived from ‘Daewonkong’ and ‘Saeolkong’.

Name of QTL	Chr. (LG) ^1^	Year	Drying Time (Hour)	Position (cM)	Physical Interval ^2^ (bp)	LOD ^3^	PVE ^4^ (%)	Add ^5^	No. Candidate Genes	References
*qPS-DS16-1*	16 (J)	Combined ^6^	24	61	7,325,202–7,760,393	5.4	11.2	−7.1	41	Novel
*qPS-DS16-2*	16 (J)	2016	48	45	29,857,920–30,146,414	19.5	44.8	−28.7	20	*qPDH1* (Funatsuki et al. 2008)
		2016	72		29,857,920–30,146,414	18.0	42.4	−28.3		
		2017	24		29,857,920–30,146,414	14.6	36.0	−20.2		
		2017	48		29,857,920–30,146,414	56.0	61.2	−40.9		
		2017	72		29,857,920–30,146,414	60.0	63.0	−41.9		
		Combined	24		29,857,920–30,146,414	19.2	48.7	−14.8		
		Combined	48		29,857,920–30,146,414	40.9	70.3	−26.8		
		Combined	72		29,857,920–30,146,414	40.8	71.8	−26.9		
*qPS-DS16-3*	16 (J)	2018	24	43	30,144,267–30,580,464	15.2	56.8	−24.4	25	*qPDH1* (nearby)
		2018	48		30,144,267–30,580,464	31.8	80.1	−37.0		
		2018	72		30,144,267–30,580,464	27.2	76.4	−34.3		

^1^ Chromosome (Linkage group). ^2^ Based on the physical position of ‘Williams82’ ver. 2.1 (www.soybase.org). ^3^ Logarithm of odds. ^4^ Phenotypic variance explained by QTL. ^5^ Additive effect; negative values indicate that ‘Daewonkong’ contributed to the trait. ^6^ Average value of 3 years, 2016, 2017, and 2018.

**Table 4:** Difference between the phenotypic data according to the allele patterns in the selected quantitative trait loci.

Genotype ^1^		*qPS-DS16-1*			*qPS-DT11*			*qPS-DT14*		
Major	Minor	24 h	48 h	72 h	24 h	48 h	72 h	24 h	48 h	72 h
P1	P1	2.5	6.5	10.9	2.6	6.5	10.7	3.5	8.1	12.7
	P2	6.2	12.5	18.6	4.3	10.7	15.6	3.8	9.0	13.6
t-value ^2^		−1.68 ^*^	−1.50 ^ns^	−1.61 ^ns^	−1.15 ^ns^	−1.45 ^ns^	−1.44 ^ns^	−0.15 ^ns^	−0.29 ^ns^	−0.24 ^ns^
P2	P1	24.3	60.5	76.1	37.2	70.3	82.8	32.0	65.6	81.7
	P2	37.6	69.9	83.5	31.9	65.9	80.8	36.8	69.8	81.6
t-value		−3.81 ^**^	−2.07 ^*^	−2.00 ^*^	1.43 ^ns^	1.12 ^ns^	0.73 ^ns^	−1.32 ^ns^	−1.09 ^ns^	0.04 ^ns^

^1^ ‘P1’ indicates that the allele patterns are the same as ‘Daewonkong’ and ‘P2’ indicates that the allele patterns are the same as ‘Tawonkong’ and ‘Saeolkong’. ^2 *^ and ^**^ denote significant difference between the phenotypes by student t-test (*p* < 0.05 and *p* < 0.01, respectively), ns denotes there was no significant difference.

**Table 5:** SNP/Indel information of candidate genes found within QTL *qPS-DS16-1*.

Gene Name	Position (bp)	Reference (Williams82)	DW	TW	SO	AA Change	Description
*Glyma.16g072700*	7,335,363	-	-	12 bp deletion	12 bp deletion	Deletion	Myb-like DNA-binding domain, DNA binding
*Glyma.16g073700*	7,446,096	A	A	T	T	Tyr/Asn	Unknown
*Glyma.16g076100*	7,665,996	C	T	C	C	Glu/Lys	Rho guanyl-nucleotide exchange factor activity
*Glyma.16g076300*	7,725,602	A	C	A	A	Met/Leu	GMC oxidoreductase
*Glyma.16g076500*	7,760,100	T	C	T	T	Leu/Ser	Adaptin N terminal region
	7,760,393	A	C	A	A	Ile/Leu	
*Glyma.16g076600*	7,775,892	C	T	C	C	Glu/Lys	Cytochrome P450,
	7,775,945	T	A	T	T	Lys/Met	(+)-abscisic acid 8′-hydroxylase activity
	7,775,948	A	G	A	A	Ile/Thr	
	7,775,970	-	18 bp insertion	-	-	Insertion	
	7,776,045	C	T	C	C	Met/Ile	
	7,777,575	-	3 bp deletion	-	-	Asn/-	

DW: Daewonkong, TW: Tawonkong, SO: Saeolkong, AA: amino acid.

## Supplementary Materials

The following are available online at https://www.mdpi.com/2223-7747/9/9/1163/s1, Table S1: Linkage map construction of the RIL population derived from a crossing between ‘Daewonkong’ and ‘Tawonkong’; Table S2: Linkage map construction of the RIL population derived from a crossing between ‘Daewonkong’ and ‘Saeolkong’; Table S3: Primer information of the candidate genes used for qRT-PCR; Table S4: The description of candidate genes; Figure S1: The dried pods of the tolerant parent (Daewonkong) and two susceptible parents (Tawonkong and Saeolkong) drying after 72 h.
