# Supplementary material for: QTL Mapping and Candidate Gene Analysis for Pod Shattering Tolerance in Soybean (Glycine max)
Source: Plants (Basel). 2020 Sep 8;9(9):1163. doi: 10.3390/plants9091163 (PMC7569788; doi:10.3390/plants9091163)
Supplement: Supplementary file 1 [file plants-09-01163-s001.zip › plants-913366-supplementary(revision).docx]

**Supplementary Table S1** Linkage map construction of the RIL population derived from a crossing between ‘Daewonkong’ and ‘Tawonkong’

| **Chromosome** | **Length**  **(cM)** | **No. of total SNP marker tested** | **No. of polymorphic marker** | **No. of marker mapped** | **Average distance between markers (cM)** |
| --- | --- | --- | --- | --- | --- |
| 1 | 128 | 8,935 | 1,524 | 115 | 1.1 |
| 2 | 171 | 10,224 | 1,442 | 172 | 1.0 |
| 3 | 108 | 8,417 | 1,136 | 110 | 1.0 |
| 4 | 133 | 8,638 | 599 | 105 | 1.3 |
| 5 | 131 | 8,024 | 669 | 95 | 1.4 |
| 6 | 124 | 9,906 | 1,460 | 98 | 1.3 |
| 7 | 118 | 8,588 | 1,363 | 110 | 1.1 |
| 8 | 181 | 10,996 | 947 | 129 | 1.4 |
| 9 | 111 | 8,996 | 1,249 | 114 | 1.0 |
| 10 | 129 | 9,309 | 1,148 | 103 | 1.3 |
| 11 | 145 | 8,412 | 990 | 98 | 1.5 |
| 12 | 100 | 7,708 | 1,220 | 85 | 1.2 |
| 13 | 158 | 10,885 | 1,441 | 161 | 1.0 |
| 14 | 112 | 7,817 | 1,341 | 109 | 1.0 |
| 15 | 115 | 10,136 | 1,614 | 115 | 1.0 |
| 16 | 103 | 7,590 | 1,843 | 120 | 0.9 |
| 17 | 144 | 8,906 | 1,064 | 105 | 1.4 |
| 18 | 139 | 9,957 | 945 | 144 | 1.0 |
| 19 | 123 | 8,719 | 1,372 | 117 | 1.1 |
| 20 | 119 | 8,212 | 1,040 | 116 | 1.0 |
| **Average** | **130** | **9,019** | **1,220** | **116** | **1.1** |
| **Total** | **2,592** | **180,375** | **24,407** | **2,321** | **22.6** |

**Supplementary Table S2** Linkage map construction of the RIL population derived from crossing between ‘Daewonkong’ and ‘Saeolkong’

| **Chromosome** | **Length**  **(cM)** | **No. of total SNP marker tested** | **No. of polymorphic marker** | **No. of marker mapped** | **Average distance between markers (cM)** |
| --- | --- | --- | --- | --- | --- |
| 1 | 119 | 8,935 | 965 | 98 | 1.2 |
| 2 | 265 | 10,224 | 1,356 | 83 | 2.8 |
| 3 | 132 | 8,417 | 1,060 | 99 | 1.3 |
| 4 | 184 | 8,638 | 937 | 84 | 2.2 |
| 5 | 121 | 8,024 | 629 | 81 | 1.5 |
| 6 | 152 | 9,906 | 971 | 62 | 2.5 |
| 7 | 125 | 8,588 | 1,257 | 95 | 1.3 |
| 8 | 191 | 10,996 | 1,332 | 70 | 2.7 |
| 9 | 183 | 8,996 | 735 | 93 | 2.0 |
| 10 | 132 | 9,309 | 906 | 97 | 1.4 |
| 11 | 181 | 8,412 | 982 | 89 | 2.0 |
| 12 | 105 | 7,708 | 698 | 37 | 2.8 |
| 13 | 159 | 10,885 | 1,279 | 104 | 1.5 |
| 14 | 132 | 7,817 | 955 | 95 | 1.4 |
| 15 | 107 | 10,136 | 1,271 | 107 | 1.0 |
| 16 | 99 | 7,590 | 1,791 | 148 | 0.7 |
| 17 | 171 | 8,906 | 928 | 70 | 2.4 |
| 18 | 115 | 9,957 | 1,443 | 80 | 1.4 |
| 19 | 126 | 8,719 | 1,106 | 44 | 2.9 |
| 20 | 118 | 8,212 | 861 | 103 | 1.1 |
| **Average** | **146** | **9,019** | **1,073** | **87** | **1.8** |
| **Total** | **2,917** | **180,375** | **21,462** | **1,739** | **36.3** |

**Supplementary Table S3** Primer information of the candidate genes used for qRT-PCR

| **Gene name** | **Primer sequence (5’-3’)** | **Product size (bp)** |
| --- | --- | --- |
| *Glyma.16g072700* | F: CTCTTTCGGGTTTGGTATGAGA | 158 |
|  | R: GCAACATCATAACCCATTGTGC |  |
| *Glyma.16g076100* | F: CTTATGTGCCTTGTTTGGACAC | 242 |
|  | R: CAATGCTGATTTTGGCTTCC |  |
| *Glyma.16g076300* | F: CCCATTGTGTTCAGCACATC | 167 |
|  | R: CCTGAACAGTGACCATTGGA |  |
| *Glyma.16g076500* | F: GCAGAGACTCCAGCAAGTAGA | 205 |
|  | R: CCATCTGCCTGACTTTTGCA |  |
| *Glyma.16g076600* | F: ATGCAGGTGGTCTTTGATGG | 126 |
|  | R: CCATGCCTTGATTAGTGTGC |  |
| *GmActin* | F: CTTCAGGCATTCACGAGACA |  |
|  | R: AGAACCACCGATCCAGACAC |  |

**F: forward, R: reverse**

**
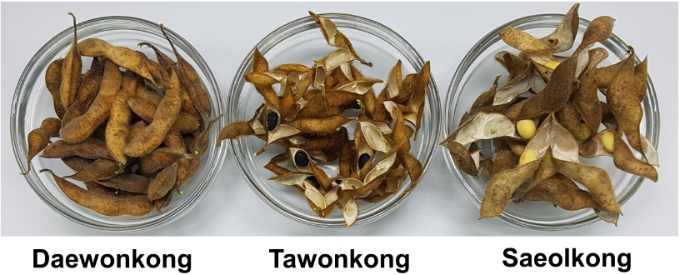
**

**Supplementary Figure S1** The dried pods of the tolerant parent (Daewonkong) and two susceptible parents (Tawonkong and Saeolkong) drying after 72 hours
